# Long-Term Survival of *Synechococcus* and Heterotrophic Bacteria without External Nutrient Supply after Changes in Their Relationship from Antagonism to Mutualism

**DOI:** 10.1128/mBio.01614-21

**Published:** 2021-08-31

**Authors:** Zenghu Zhang, Shailesh Nair, Lili Tang, Hanshuang Zhao, Zhenzhen Hu, Mingming Chen, Yao Zhang, Shuh-Ji Kao, Nianzhi Jiao, Yongyu Zhang

**Affiliations:** a Key Laboratory of Biofuels, Shandong Provincial Key Laboratory of Energy Genetics, Qingdao Institute of Bioenergy and Bioprocess Technology, Chinese Academy of Sciences, Qingdao, China; b University of Chinese Academy of Sciences, Beijing, China; c Center for Ocean Mega-Science, Chinese Academy of Sciences, Qingdao, China; d State Key Laboratory of Marine Environmental Science, Xiamen University, Xiamen, China; e State Key Laboratory of Marine Resource Utilization in the South China Sea, Hainan University, Haikou, China; University of Georgia

**Keywords:** *Synechococcus*, heterotrophic bacterial community, algae-bacteria interaction, mutualism, nitrogen cycle

## Abstract

Marine phytoplankton and heterotrophic bacteria share a very close but usually changeable relationship. However, the ultimate fate of their unstable relationship on a long-term scale is unclear. Here, the relationship between *Synechococcus* and heterotrophic bacterial communities underwent a dramatic shift from antagonism to commensalism and eventually to mutualism during long-term cocultivation. The relationship change is attributed to the different (even opposite) effects of diverse bacterial members on *Synechococcus* and the ratio of beneficial to harmful bacteria. Different bacterial members also interact with each other (e.g., quorum-sensing communication, hostility, or mutual promotion) and drive a dynamic succession in the entire community structure that corresponds exactly to the shift in its relationship with *Synechococcus*. In the final mutualism stage, a self-sufficient nitrogen cycle, including nitrogen fixation, denitrification, and organic nitrogen degradation, contributed to the healthy survival of *Synechococcus* for 2 years without an exogenous nutrient supply. This natural selective trait of *Synechococcus* and heterotrophic bacteria toward mutualism under long-term coexistence provides a novel clue for understanding the ubiquity and competitive advantage of *Synechococcus* in global oceans.

## INTRODUCTION

Phytoplankton (including cyanobacteria) and heterotrophic bacteria are closely related and are important regulators of the ocean ecosystem (1). As the dominant primary producers (phytoplankton) and drivers of biogeochemical cycles (bacteria) in the ocean (2, 3), they interact with each other and underpin most functions of marine ecological processes (4–6). The interactions between phytoplankton and heterotrophic bacteria are multifarious, spanning from mutualism (7) to antagonism (8) or from cooperation (9) to allelopathy (10). These interactions have recently been thoroughly examined in several excellent reviews (1, 11, 12). However, despite being scrutinized for at least half a century, the “black box” of the highly complex relationship between phytoplankton and heterotrophic bacteria is not well uncovered (12, 13).

Indeed, the relationship between phytoplankton and heterotrophic bacteria is usually unstable and can change under the influence of environmental and/or biological factors. For example, during algal blooms, the effect of the bacterial community on phytoplankton can shift from promoting algal growth or symbiosis with algae to antagonizing algae (14–17). In the long-term cocultivation of a heterotrophic bacterium, *Tropicibacter* sp., and *Synechococcus* sp. strain WH7803, the bacterial effect on *Synechococcus* shifted from weakly maintaining the growth of *Synechococcus* to promoting its high-density growth, which was likely driven by nutrient recycling (18). In contrast, a mutualist-to-parasite shift occurred in the cocultivation of phytoplankton and *Roseobacter*, which depended on the growth state of the phytoplankton and the concomitant changes in substances released from the bacterium (19, 20). Although these two cases, to a certain extent, reflect the complex and dynamic phytoplankton-bacterium interactions, they focused on a single bacterium’s effect on phytoplankton. In the natural environment, complex multispecies communities rather than a single bacterium interact with the phytoplankton. In this sense, it is more important to consider the role of bacterial communities, not just individual bacterial species, while investigating the relationship between phytoplankton and heterotrophic bacteria.

The most abundant constituents of phytoplankton are the picocyanobacteria, dominated by the genera *Synechococcus* and *Prochlorococcus*. *Synechococcus* is widely distributed in various marine environments, including coastal, pelagic, and polar oceans, etc. (21, 22). Despite their small size (diameter of <2 μm), over 40% of *Synechococcus* cells were found to be conjoint with other bacteria under *in situ* observation (23). Moreover, the metagenomic information collected during the *Tara* Oceans expedition indicated that the close relationship between heterotrophic bacteria and *Synechococcus* in oligotrophic oceans has an important role in carbon export processes (24). However, to date, the relationship between *Synechococcus* and heterotrophic bacteria at the community level has been poorly understood. Here, the dynamic relationship between *Synechococcus* sp. strain PCC7002 and a natural seawater bacterial community during a long-term cocultivation process was investigated. Intriguingly, we observed that their relationship changed dramatically, after which *Synechococcus* could survive permanently without added nutrients. Here, we unveil the underlying drivers of this phenomenon, which provides novel insight into the interactions between phytoplankton and heterotrophic bacteria in the ocean.

## RESULTS

### Dynamic relationships between *Synechococcus* and the bacterial community during long-term cocultivation.

During long-term cocultivation, the relationship between *Synechococcus* sp. PCC7002 and the coexisting heterotrophic bacterial community changed dramatically from antagonism to commensalism and finally to mutualism.

**FIG 1:**
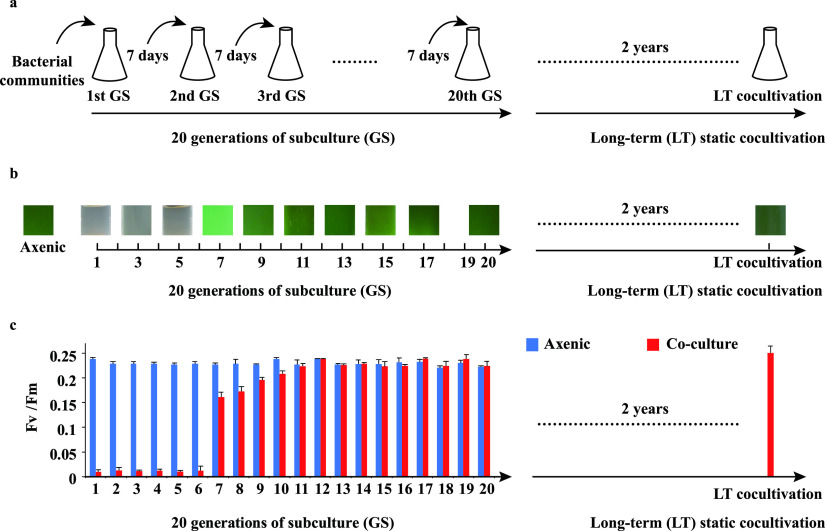
Serial subculture and long-term static cocultivation of *Synechococcus* and the natural bacterial community. (a) The *Synechococcus*-bacteria cocultivation system was initially established by adding seawater bacterial communities to an axenic culture of *Synechococcus* sp. PCC7002 to be cocultivated for 7 days (i.e., the 1st GS). Next, the bacterial community from the 1st GS was added to another fresh axenic *Synechococcus* culture to generate the 2nd GS over another 7 days. Subculturing was carried out based on the above-described steps for a total of 20 generations. Thereafter, the 20th GS was transferred to large-volume fresh axenic *Synechococcus* cultures for 2 years of static cocultivation. (b) Color changes of the cocultivation system of *Synechococcus* and the natural bacterial community in different GSs. (c) Responses of the *F_v_*/*F_m_* of *Synechococcus* to the coexisting bacterial community during serial subculture and the long-term static cocultivation process.

Upon inoculation of a natural bacterial community to an exponentially growing *Synechococcus* culture (i.e., the first generation of the subculture [1st GS]), the bacterial community showed a strong growth-inhibitory or algicidal effect on *Synechococcus*, which was reflected by the obvious color change in the cocultivation system from healthy green to colorless with a white deposition (Fig. 1b). The *Synechococcus* abundance in the cocultivation system also decreased significantly from 1.7 × 10^5^ ± 0.14 × 10^5^ to 5.0 × 10^4^ ± 0.36 × 10^4^ cells ml^−1^, while that in the bacterium-free control set increased from 1.7 × 10^5^ ± 0.02 × 10^5^ to 2.5 × 10^5^ ± 0.01 × 10^5^ cells ml^−1^. This inhibitory effect persisted until the 6th GS. We have also confirmed that no viral infection was involved in causing the decrease in the abundance of *Synechococcus* (see Text S1 and Fig. S1 in the supplemental material). The photosystem II maximum quantum yield (*F_v_*/*F_m_*) of *Synechococcus* remained as low as ca. 0.01 during the first 6 generations, while that in the control set was far higher (ca. 0.22) (Fig. 1c). Therefore, the first 6 GSs were called the antagonism stage of the relationship.

10.1128/mBio.01614-21.1 TEXT S1 Supplemental materials and methods, results, figure legends, and references. Download Text S1, DOC file, 0.06 MB. Copyright © 2021 Zhang et al. 2021 Zhang et al. https://creativecommons.org/licenses/by/4.0/ This content is distributed under the terms of the Creative Commons Attribution 4.0 International license.

10.1128/mBio.01614-21.2 FIG S1 Time course of virus and *Synechococcus* abundances in the first generation of the subculture. Download FIG S1, TIF file, 1.3 MB. Copyright © 2021 Zhang et al. 2021 Zhang et al. https://creativecommons.org/licenses/by/4.0/ This content is distributed under the terms of the Creative Commons Attribution 4.0 International license.

Thereafter, from the 7th to the 11th GSs, the bacterial community’s inhibitory effect on *Synechococcus* gradually weakened, and the cocultivation system was no longer clear but light green. The *F_v_*/*F_m_* also gradually increased from ca. 0.15 to 0.2 and reached the same level as that of axenic *Synechococcus* in the 11th GS. From the 11th GS onwards, there was little difference between the colors of the cocultivation system and the control set. The bacterial community no longer suppressed *Synechococcus* growth, and the *F_v_*/*F_m_* value was almost equal to that of the control set. This behavior continued until the end of the serial subculturing process (the 20th GS). Therefore, the stage from the 7th to the 20th GSs was called the commensalism stage. In the last four subcultures (i.e., the 17th to the 20th GSs), the *F_v_*/*F_m_* of *Synechococcus* was even slightly higher than that of the control set.

From then on, the coexisting bacterial community was transferred to a culture of exponentially growing *Synechococcus* for long-term observations. Interestingly, even after 2 years without any additional supply of nutrients, *Synechococcus* continued to grow healthily in the static cocultivation system, maintaining a green color. The *F_v_*/*F_m_* of *Synechococcus* was ca. 0.25, comparable to the *F_v_*/*F_m_* values of healthy axenic *Synechococcus* PCC7002 (25) and other cyanobacteria (26). In contrast, axenic *Synechococcus* in the control set (without additional N source replenishment) collapsed and turned colorless with a white deposition within 3 months. It should be mentioned that during the long-term cocultivation process, the coculture was not contaminated by any other phytoplankton, and *Synechococcus* was confirmed to be identical to the original *Synechococcus* sp. PCC7002 (Text S1). This indicated that a mutually beneficial interaction persisted between *Synechococcus* and the coexisting bacterial community, and this stage was called the mutualism stage.

### Succession of bacterial community structure during long-term cocultivation.

**FIG 2:**
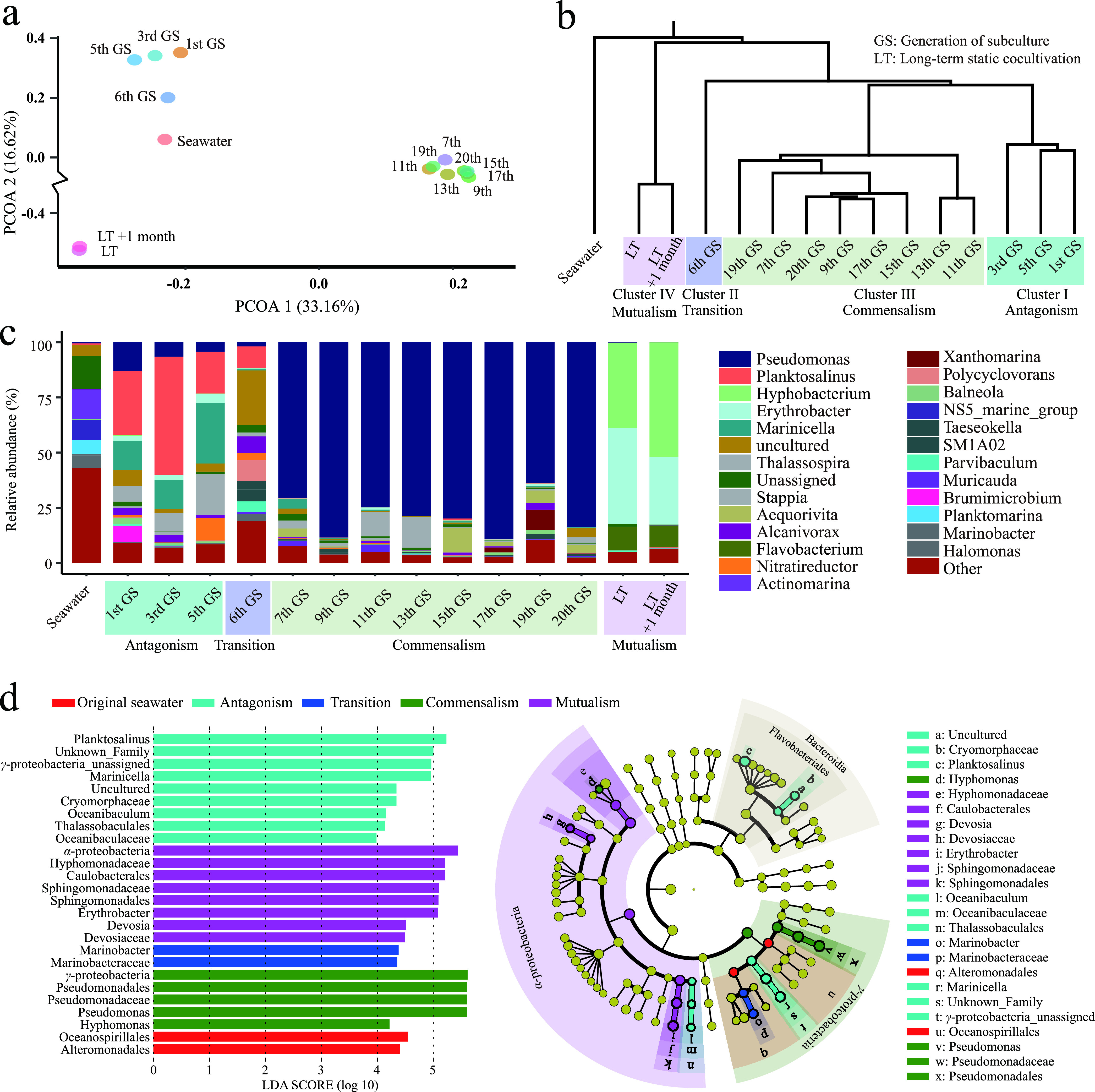
Dynamic changes in the bacterial community structure during long-term cocultivation with *Synechococcus*. Constrained principal-coordinate analysis (PCoA) based on the Bray-Curtis distance matrix (a), hierarchical clustering based on the Bray-Curtis distance matrix (b), bacterial community structure at the genus level (c), and dominant bacterial taxa with significant differences in different stages/clusters based on LEfSe analysis (LDA score of >2; *P* < 0.05) (d) are shown.

Throughout the cocultivation process, the bacterial communities changed dynamically, showing obvious succession characteristics. The changing bacterial communities can be clustered into four distinct groups (i.e., clusters I to IV) according to principal-coordinate analysis (PCoA) and hierarchical clustering analysis (Fig. 2a and b). Cluster I included the bacterial community profiles from the 1st, 3rd, and 5th GSs (Fig. 2b), all of which had a strong inhibitory effect on *Synechococcus* growth and corresponded exactly to the antagonism stage of their relationship with *Synechococcus*. Notably, in the antagonism stage, *Planktosalinus* and *Marinicella* were the most dominant genera, with relative abundances accounting for 18.9 to 53.6% and 13.2 to 27.5% of the whole bacterial community, respectively (Fig. 2c and d). The 6th GS, a transitional phase after which the growth-inhibitory effect of the bacterial community started to diminish, was in cluster II (Fig. 2b), with *Polycyclovorans* taking up the position of *Marinicella* as the second most abundant group. All the bacterial community profiles from the 7th to the 20th GSs were in cluster III (Fig. 2b), which corresponds exactly to the commensalism stage of the relationship with *Synechococcus*. Here, Pseudomonas became the most dominant bacterial group, with a relative abundance of 63.8 to 89.1% (Fig. 2c). Concurrently, the relative abundance of the genera *Planktosalinus*, *Marinicella*, and *Polycyclovorans* decreased to less than 1%. The bacterial community from the long-term static cocultivation system was found to be highly curated in cluster IV (Fig. 2b), corresponding exactly to the mutualism stage of its relationship with *Synechococcus*. In the mutualism stage, the genera *Erythrobacter* and *Hyphobacterium* predominated the bacterial profiles, with a total relative abundance of >80%.

### Effect of the bacterial individuals from the cocultivation system on *Synechococcus* growth.

**FIG 3:**
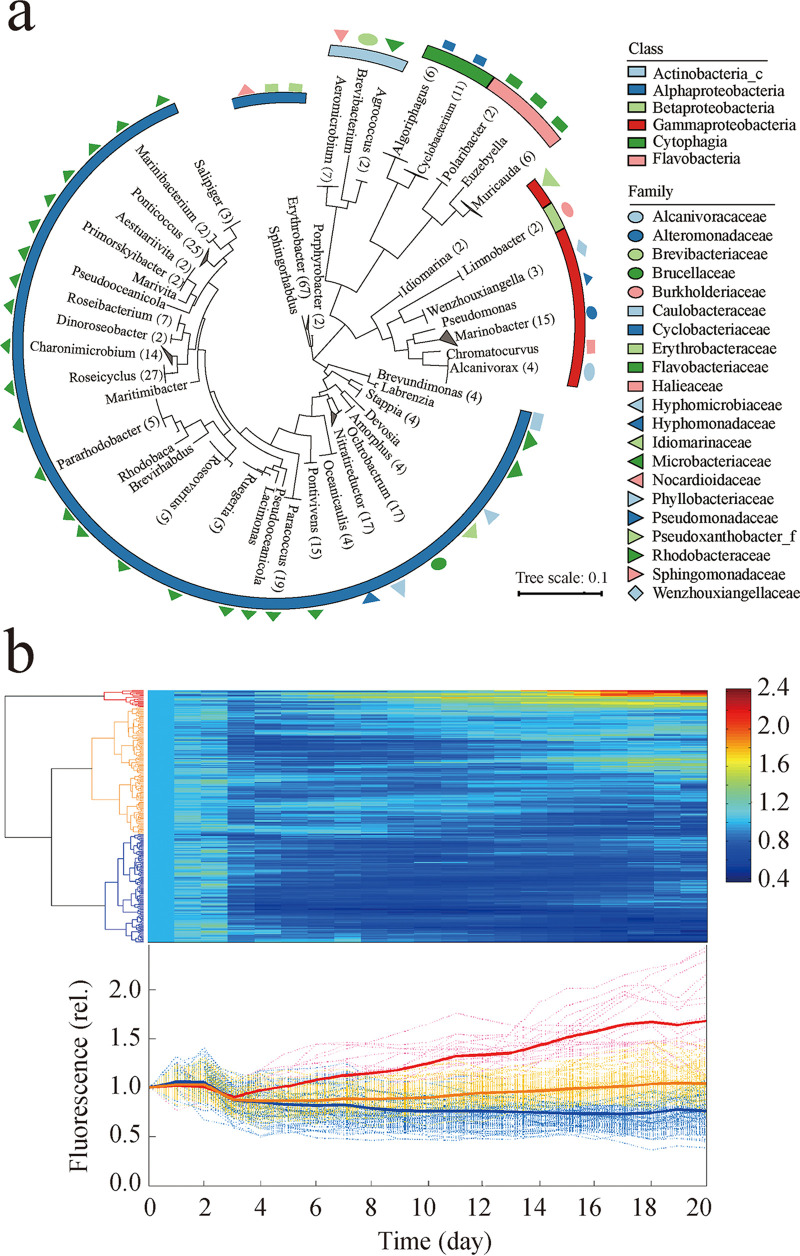
Classification of 326 bacterial individuals from the antagonism stage and their effect on the growth of *Synechococcus* sp. PCC7002. (a) Maximum likelihood tree based on 16S rRNA genes. The strains belonging to the same genus were merged into one branch, where the number of strains is shown in parentheses. (b) Effect of each bacterial individual on the growth (using fluorescence as an indicator) of *Synechococcus* sp. PCC7002. Hierarchical clustering and a heat map were established based on the normalized fluorescence values and normalized fluorescence curves of *Synechococcus-*bacterium cocultures. These values were normalized against those of the axenic control. A total of 46.0% (150 out of 326) of the bacterial individuals had remarkable negative effects on *Synechococcus* growth (blue), 26.1% (85 out of 326) promoted the growth of *Synechococcus* (red), and the others had no obvious effects (yellow).

Totals of 326 and 938 bacterial strains were isolated from the antagonism stage (represented by the 3rd GS) and the mutualism stage (i.e., long-term static cocultivation), respectively. The 326 strains from the antagonism stage belonged to 45 genera and 56 species (Fig. 3a; see also Table S1 at https://doi.org/10.6084/m9.figshare.15059517.v3), while the 938 strains from the mutualism stage had relatively low diversity, belonging to 25 genera and 34 species (see Table S2 at the URL mentioned above).

Interestingly, although the bacterial community in the antagonism stage showed a strong growth-inhibitory or algicidal effect on *Synechococcus*, among the 326 isolated bacterial strains, only 150 (46.0%) suppressed the growth of *Synechococcus*. In contrast, 85 out of these 326 bacterial strains (i.e., 26.1%) promoted the growth of *Synechococcus*. The remaining 91 strains (27.9%) had no obvious effects on *Synechococcus* (Fig. 3b; see also Table S3 at https://doi.org/10.6084/m9.figshare.15059517.v3). Similarly, although all bacterial communities in the mutualism stage showed a mutualistic relationship with *Synechococcus*, among the representative bacterial strains of all 34 different species, 1 bacterial strain (i.e., Microbacterium oxydans SN037) inhibited the growth of *Synechococcus*, and 14 strains (41.2%) promoted the growth of *Synechococcus* (see Table S3 at the URL mentioned above). Overall, the isolated bacterial community from the antagonism stage was composed of a higher proportion of inhibitory bacteria (46.0%) than beneficial bacteria (26.1%), while that isolated from the mutualism stage had more beneficial bacteria (ca. 41.2%) (see Table S3 at the URL mentioned above).

### Interactions between different members of the bacterial community.

**FIG 4:**
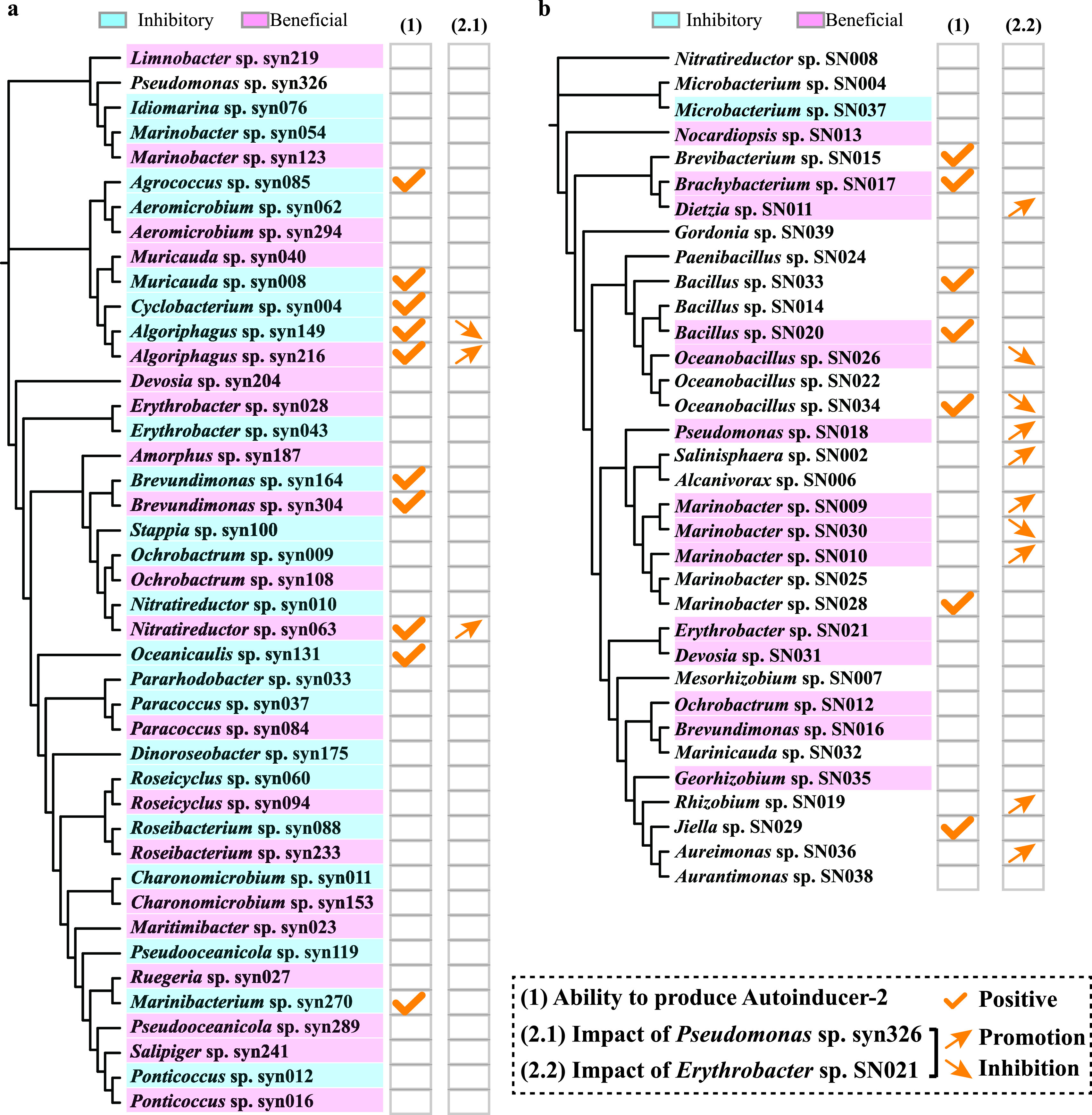
Interactions between different members of the bacterial community. The ability of 42 (a) and 34 (b) representative bacterial individuals from the antagonism and mutualism stages, respectively, to produce autoinducer-2 and the impact of the two tested strains (*Erythrobacter* sp. SN021 and Pseudomonas sp. syn326) on these representative bacteria are shown.

Forty-two and 34 bacterial strains, each as a representative strain of a different genus or species from the antagonism and mutualism stages, respectively, were tested for their quorum-sensing (QS) activity. Approximately 24% (10 of 42) of the representative strains from the antagonism stage and 20% (7 of 34) from the mutualism stage showed the ability to produce the QS signal molecule autoinducer-2 (AI-2) (Fig. 4), but the effect of different bacteria on *Synechococcus* growth was independent of their AI-2-producing capability. In addition, the bacterial communities coexisting with *Synechococcus* during the serial subculturing process were predicted to be involved in various quorum-sensing processes (Fig. S2; see also Table S4 at https://doi.org/10.6084/m9.figshare.15059517.v3), e.g., competence, motility, and iron uptake regulated by acylated homoserine lactones (AI-1), aromatic signaling molecules, or diffusible signal factors.

10.1128/mBio.01614-21.3 FIG S2 Distribution of quorum-sensing genes in each cocultivation system. PICRUSt2 was applied for the predictions of microbial functional genes. Download FIG S2, TIF file, 1.7 MB. Copyright © 2021 Zhang et al. 2021 Zhang et al. https://creativecommons.org/licenses/by/4.0/ This content is distributed under the terms of the Creative Commons Attribution 4.0 International license.

We also tested the effects of two representative strains, i.e., Pseudomonas sp. strain syn326 and *Erythrobacter* sp. strain SN021, on other isolates via plate assays. These two strains were phylogenetically similar to the most-abundant amplicon sequence variants (ASVs) in the commensalism and mutualism stages, i.e., Pseudomonas-like ASV_1 and *Erythrobacter*-like ASV_3 (see Table S5 at the URL mentioned above). Although Pseudomonas sp. syn326 did not influence the growth of *Synechococcus*, it inhibited the bacterium *Algoriphagus* sp. syn149, a strain with an inhibitory effect on *Synechococcus*, and promoted the growth of *Nitratireductor* sp. syn063 and *Algoriphagus* sp. syn216, which were both beneficial to *Synechococcus* (Fig. 4 and Fig. S3). *Erythrobacter* sp. SN021 was beneficial to *Synechococcus* growth and exhibited a strong ability to regulate the growth of other bacteria (Fig. 4 and Fig. S3). For instance, it inhibited the growth of *Marinobacter* sp. SN030 but promoted that of *Marinobacter* sp. SN009.

10.1128/mBio.01614-21.4 FIG S3 Bacterium-bacterium interactions. The inhibition zone around the agar slab containing Pseudomonas sp. syn326 (blue arrow) or *Erythrobacter* sp. SN021 (black arrow) indicates their inhibitory effect on the growth of the tested strains. The promotion zone indicates the beneficial effect. Download FIG S3, TIF file, 2.8 MB. Copyright © 2021 Zhang et al. 2021 Zhang et al. https://creativecommons.org/licenses/by/4.0/ This content is distributed under the terms of the Creative Commons Attribution 4.0 International license.

### Rich inorganic nitrogen in the mutualism stage and nitrogen metabolism genes in the bacterial communities.

We determined the concentrations of different inorganic nitrogen compounds that may significantly affect the growth of *Synechococcus* in the mutualism stage and in the axenic control set (see Table S6 at https://doi.org/10.6084/m9.figshare.15059517.v3). In the control set containing only exponentially growing axenic *Synechococcus* cells, the concentrations of nitrate, nitrite, and ammonium were 8,015.3 ± 808.2 μmol/liter, 2.5 ± 0.4 μmol/liter, and 2,682.1 ± 236.4 μmol/liter, respectively, which were mainly from the fresh A^+^ medium. Surprisingly, in the mutualism stage, although no nutrients had been artificially supplemented for 2 years, the inorganic nitrogen in the cocultivation system remained at high levels (i.e., nitrate at 4,558.2 ± 677.2 μmol/liter, nitrite at 72.5 ± 12.4 μmol/liter, and ammonium at 2,277.5 ± 153.2 μmol/liter). As the host *Synechococcus* sp. PCC7002 cannot fix nitrogen (see Tables S7 and S8 at the URL mentioned above) (27, 28), and we did not artificially supplement N nutrients, the rich nitrogen source in the mutualism stage is a mystery, and it is speculated to come from the contribution of coexisting bacterial communities. To verify this hypothesis, we tested the 34 representative bacterial strains isolated from the mutualism stage to determine whether they contain the nitrogenase gene *nifH*. As a result, four strains (i.e., *Rhizobium* sp. SN019, *Georhizobium* sp. SN035, *Mesorhizobium* sp. SN007, and *Bacillus* sp. SN014) were found to be positive for the *nifH* gene, among which *Mesorhizobium* sp. SN007 and *Bacillus* sp. SN014 also showed actual nitrogenase activity in the acetylene reduction assay. Meanwhile, in the metagenome of the bacterial community at the mutualism stage, in addition to the nitrogen-fixing gene (*nifH*) that catalyzes nitrogen fixation, there are many other key genes involved in different nitrogen metabolism processes, i.e., organic nitrogen degradation, dissimilatory/assimilatory nitrate reduction, and denitrification (Fig. S4; see also Table S9 at the URL mentioned above). Among them, the most abundant genes were *gdh* (glutamate dehydrogenase) and *gs* (glutamate synthase), which were related to the conversion of *Synechococcus*-derived organic nitrogen to inorganic nitrogen (i.e., ammonia production). The *narGHIJ* genes, encoding nitrate reductases involved in dissimilatory nitrate reduction and nitrate-to-nitrite conversion, were also abundant in the mutualism stage. Nitrite can be further reduced to ammonium by the nitrite reductase encoded by the *nrfA* gene in the metagenome, which would support the growth of *Synechococcus* (Fig. S4). In addition, there was also a relatively high proportion of genes in the metagenome participating in denitrification, including the *nirSK* (nitrite reductase), *norBC* (nitric oxide reductase), and *nosZ* (nitrous oxide reductase) genes, which potentially induced the loss of nitrogen. Therefore, nitrogen input (i.e., nitrogen fixation) was particularly important to offset the possible nitrogen loss and maintain the high nitrogen concentration in the long-term mutualism stage of the coculture system.

10.1128/mBio.01614-21.5 FIG S4 Genes involved in the nitrogen cycle in the heterotrophic bacterial community cocultured with *Synechococcus* during the mutualism stage. The numbers in brackets beside each gene represent the abundances for the gene or gene family. Download FIG S4, TIF file, 1.0 MB. Copyright © 2021 Zhang et al. 2021 Zhang et al. https://creativecommons.org/licenses/by/4.0/ This content is distributed under the terms of the Creative Commons Attribution 4.0 International license.

## DISCUSSION

Phytoplankton and heterotrophic bacterium relationships are changeable and depend highly on many aspects, including external environmental and biotic factors. However, the underlying mechanisms of the relationship variability between phytoplankton and heterotrophic bacteria remain unclear. Here, we experimentally cocultivated a natural bacterial community with *Synechococcus* sp. PCC7002 and tracked the dynamics of their relationship on a long-term scale. We found that the relationship between the *Synechococcus* and heterotrophic bacterial communities underwent a dramatic shift from antagonism to commensalism and finally to mutualism (Fig. 1b and c). Concurrently, during this process, the bacterial communities also showed succession characteristics (clusters I to IV) (Fig. 2a and b) that were mapped to the timeline of the shift of the *Synechococcus*-bacterium relationship from antagonism to mutualism. This suggested that the succession of bacterial communities might be an important factor boosting the transition of the relationship between *Synechococcus* and the bacterial community.

Cluster I (Fig. 2a and b), representing the antagonistic stage (i.e., 1st through 5th GSs), was dominated by the genera *Planktosalinus* and *Marinicella* (Fig. 2c and d). These two bacterial genera are affiliated with *Bacteroidetes* and *Gammaproteobacteria*, respectively, which are frequent inhabitants of the algal phycosphere (12, 29, 30) and are known to include many algicidal bacteria (8, 31). In addition, *Bacteroidetes* or *Gammaproteobacteria* often increase significantly in the late algal bloom period (32–34). Similarly, the bacterial community in cluster I showed high relative abundances of *Gammaproteobacteria* (27 to 35%) and *Bacteroidetes* (25 to 57%) (Fig. 2c; see also Fig. S5 in the supplemental material). In this sense, the relatively high proportion of inhibitory bacteria may be the driving force behind the decline in *Synechococcus* at this stage. This was validated by the effects of bacterial individuals isolated from the cocultivation system on the growth of *Synechococcus*. As expected, nearly half of the bacterial individuals (46.0%) from the antagonism stage inhibited the growth of *Synechococcus*.

10.1128/mBio.01614-21.6 FIG S5 Dynamic changes in the bacterial community structure at the phylum level during long-term cocultivation with *Synechococcus*. Download FIG S5, TIF file, 1.7 MB. Copyright © 2021 Zhang et al. 2021 Zhang et al. https://creativecommons.org/licenses/by/4.0/ This content is distributed under the terms of the Creative Commons Attribution 4.0 International license.

From the 7th GS until the last (20th) GS of the serial subculturing process, no sign of *Synechococcus* growth inhibition was observed, suggesting that the antagonistic effect of the bacterial community diminished, giving rise to the emergence of the commensalism stage. The bacterial community structure profiles significantly differed from those of the previous stage (Fig. 2d), with a surge in Pseudomonas accounting for 60 to 90% of the total bacterial community (Fig. 2c). The ability of Pseudomonas species to promote the growth of host algae by itself or by regulating the growth of other bacteria has been reported. For example, cocultivation of the alga Chlorella vulgaris with a Pseudomonas strain yielded a 1.4-times-higher algal cell concentration than with axenic C. vulgaris (35). Additionally, some Pseudomonas spp. can also produce bacteriocins (peptidic toxins with antibacterial activity) and antibiotics (such as pyrrolnitrin, pyoluteorin, and 2,4-diacetylphloroglucinol) to inhibit other bacteria (29). Two representative strains of Pseudomonas (i.e., Pseudomonas species strains syn326 and SN018) isolated in this study (Fig. 4; see also Table S5 at https://doi.org/10.6084/m9.figshare.15059517.v3) had no inhibitory effect on the growth of *Synechococcus*, and in contrast, Pseudomonas sp. SN018 significantly promoted the growth of *Synechococcus* (Fig. 4). Although Pseudomonas sp. syn326 did not have a direct growth-promoting effect on *Synechococcus*, it inhibited the growth of some inhibitory bacteria (e.g., *Algoriphagus* sp. syn149) and promoted the growth of some beneficial bacteria (e.g., *Nitratireductor* sp. syn063 and *Algoriphagus* sp. syn216) (Fig. 4a). These abilities of Pseudomonas might have favored its proliferation, directly or indirectly benefiting *Synechococcus* growth and driving the establishment of a commensalism relationship between *Synechococcus* and the heterotrophic bacterial community.

The improved *F_v_*/*F_m_* of *Synechococcus* in the cocultivation system, especially in the last few generations (from the 17th to the 20th GSs) (Fig. 1c), suggested a positive interaction between *Synechococcus* and the heterotrophic bacterial community. Similar phenomena have also been observed by Motone et al. (36). Here, we asked how long this relationship could be maintained if there were no further subculture steps. Therefore, we conducted an additional static cocultivation experiment for 2 years. Interestingly, without any additional supplement of nutrients, *Synechococcus* maintained a healthy growth state for 2 years. In contrast, the axenic *Synechococcus* culture in the control set collapsed within 3 months. This indicates that a mutualistic relationship between *Synechococcus* and the bacterial community was formed in the cocultivation system. During the long-term mutualism stage, *Hyphomonadaceae* (comprising *Hyphobacterium*) and *Erythrobacter* were the most abundant taxa in the bacterial community. Most species of the *Hyphomonadaceae* can attach to or form biofilms and have the potential to degrade complex organic compounds (37). These characteristics may help them become the dominant bacterial group coexisting with the host *Synechococcus*. Moreover, *Hyphomonadaceae* have often been isolated from dinoflagellates and diatoms (38), suggesting an unknown close relationship between phytoplankton and *Hyphomonadaceae*. *Erythrobacter* also tends to inhabit the phycosphere environment (1, 39, 40) and can affect the growth of other bacteria. For instance, upon exposure to polyunsaturated aldehydes (PUAs), algal cell exudates with antimicrobial activity, most bacteria, but not *Erythrobacter*, were inhibited (41). Members of the *Erythrobacter* genus also have strong signaling molecule-producing capabilities and can regulate other bacterial interactions by quorum quenching (42, 43). Here, it was verified that the isolated representative strain of the genus *Erythrobacter* in the mutualism stage, namely, *Erythrobacter* SN021, can promote the growth of *Synechococcus* (Fig. 4b). In addition, 16 metagenome-assembled bacterial genomes (bin.1 to -16) (see Table S10 at https://doi.org/10.6084/m9.figshare.15059517.v3) were obtained from the mutualism stage, including a metagenome-assembled genome classified as *Erythrobacter* (i.e., bin.3). Among them, 12 strains possessed 4 to 7 auxin synthesis genes (see Table S10 at the URL mentioned above). Auxins are a class of phototrophic hormones, and it has been reported that auxins (especially indole-3-acetic acid, the most abundant auxin) have a growth-stimulating effect on phytoplankton (44–46). Furthermore, among all 34 different bacterial species from the mutualism stage, more than 40% significantly promoted the growth of *Synechococcus*, and only 1 had an inhibitory effect on the growth of *Synechococcus* (see Table S3 at the URL mentioned above). Overall, we speculated that the rise of beneficial bacteria in the bacterial community aids in maintaining the long-term mutualistic relationship between *Synechococcus* and the bacterial community at this stage.

We recognized that the final effect of the bacterial community on the host *Synechococcus* may not be the result of simply the accumulation or offset of the effects of different individual bacteria but could also be concomitant with close interactions among bacteria. It was roughly estimated that more than 20% of the strains isolated from the antagonism or mutualism stage had the ability to produce autoinducer-2 (Fig. 4). It was also predicted that bacterial communities may have additional forms of quorum-sensing communication throughout the serial subculture and static cocultivation processes, which would indicate that bacterium-bacterium interactions were common in these bacterial communities (Fig. S2). Besides, Pseudomonas sp. syn326 can differentially affect the growth of two strains with quorum-sensing activity but with different effects on *Synechococcus* growth (Fig. 4a), indicating that the bacterial interaction can spread through a cascade effect, forming a potential interaction network and likely driving the succession of the entire bacterial community structure during long-term cocultivation.

Nitrogen availability is an important growth-limiting factor for picocyanobacteria (47). *Synechococcus* sp. PCC7002 cannot fix nitrogen by itself (see Tables S7 and S8 at https://doi.org/10.6084/m9.figshare.15059517.v3) (27, 28); the unexpectedly high concentration of inorganic nitrogen (e.g., NO_3_^−^ and NH_4_^+^) in the mutualistic cocultivation system implies that the bacterial community likely played a crucial role in providing available inorganic nutrients. Here, we did find evidence of the nitrogen-cycling genetic potential of the bacterial community via metagenomic analysis (Fig. S4; see also Table S9 at the URL mentioned above), including organic nitrogen mineralization, nitrogen fixation, and dissimilatory nitrate reduction. Previously, it was reported that a single bacterium can maintain the growth of *Synechococcus* for 200 days through nutrient recycling (i.e., mineralization) (18). Here, the abundant organic nitrogen degradation genes (e.g., *gdh*) found in the metagenomes of the long-term cocultivation system suggest that the bacterial community can drive the transformation of nitrogen from organic to inorganic forms during growth depending on the organic matter (containing organic nitrogen) released by *Synechococcus* (see Table S9 at the URL mentioned above) (48, 49), which in turn can support the growth of *Synechococcus*. Moreover, the abundant denitrification genes (*nirS*, *nirK*, *norC*, *norB*, and *nosZ*) found in the metagenomes implicate the potential of the bacterial community to cause nitrogen loss (Fig. S4). This hints that there must be a nitrogen replenishment process (e.g., nitrogen fixation) to compensate for the loss of nitrogen caused by denitrification during the mutualism stage and balancing nitrogen cycling. As expected, we validated the nitrogen-fixing gene (*nifH*) and nitrogenase activity of the bacterial individuals isolated from the mutualism stage and observed the potential nitrogen-fixing activity of four strains among them, further supporting our speculation that nitrogen-fixing activities may be a driving force for nitrogen supply in the mutualism stage. In addition, it has been reported that the growth of phytoplankton can be inhibited by high concentrations of NO_2_^−^ (50, 51). The comparatively high abundance of nitrate reductase genes (*narGHIJ* genes), whose products can reduce NO_3_^−^ to NO_2_^−^, may have been compensated for by the products of abundant nitrite reductase genes (*nirK* and *nirS*), nullifying the possibility of NO_2_^−^ overproduction (Fig. S4; see also Table S9 at the URL mentioned above). Thus, without artificial supplementation of any nitrogen nutrients, a self-sufficient nitrogen cycle (especially including nitrogen fixation) within the cocultivation system during the long-term mutualism stage might have supported the healthy survival of the host *Synechococcus*, and in return, the organic carbon substances synthesized by *Synechococcus* photosynthesis met the nutritional needs of the heterotrophic bacterial community. However, no genes responsible for ammonia oxidation (e.g., *amoA*) were found in the metagenomes, so how nitrate was produced and maintained at a high concentration in the long-term mutualism stage is still unclear and requires further research. In addition to the nitrogen cycle, genomic capability for the metabolic generation of phosphorus, iron, and vitamin B_12_ was also observed in the metagenome; e.g., the bacterial community may contribute to the phosphorus cycle by the mineralization of organic phosphorus and the solubilization of recalcitrant phosphorus (see Table S11 at the URL mentioned above) and to the iron cycle through siderophore production and iron acquisition and transport (see Table S12 at the URL mentioned above). Besides, the bacterial community can also synthesize vitamin B_12_ necessary for the growth of *Synechococcus* sp. PCC7002 (52) (see Table S13 at the URL mentioned above). This indicates the complexity of nutritional self-sufficiency in the cocultivation system. In addition, we admit that the bacterial community cocultured with *Synechococcus* in the laboratory will be different from that of *in situ* seawater. Meanwhile, in the *in situ* environment, the relationship between *Synechococcus* and bacterial communities will be much more complex (such as grazing or viral lysis, etc.) than what we observed in the laboratory. These need more in-depth study in the future.

**FIG 5:**
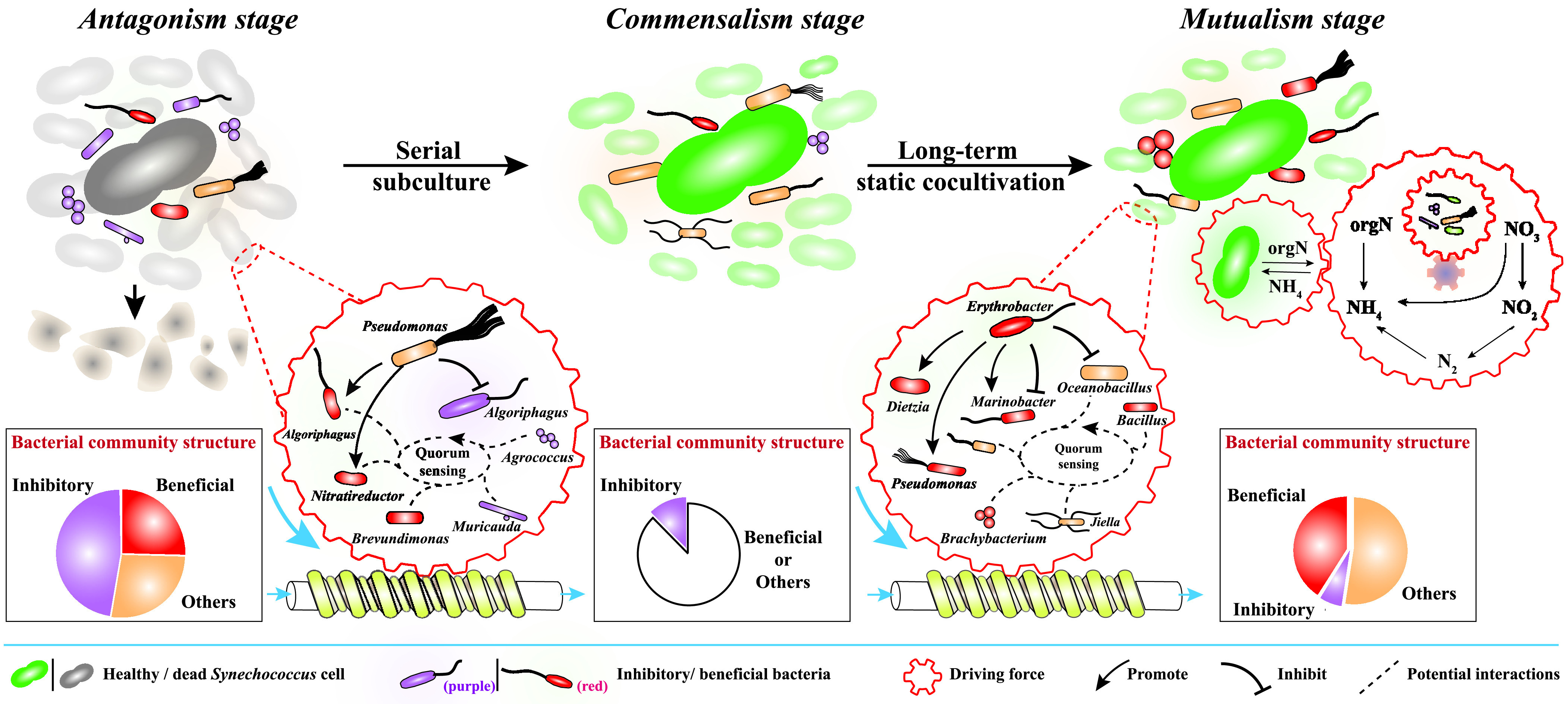
Schematic of the succession of bacterial community structure and function during long-term cocultivation with *Synechococcus*. The initial bacterial community with a high proportion of inhibitory bacteria inhibited the growth of *Synechococcus* in the antagonism stage. Under the interaction between different members of the bacterial community during serial subculture, the inhibitory bacteria were suppressed to a point that they could not completely inhibit the growth of *Synechococcus.* The relationship between *Synechococcus* and the heterotrophic bacterial community gradually entered the commensalism stage. Thereafter, as the proportion of beneficial bacteria continued to increase, a mutually beneficial relationship was established between *Synechococcus* and the heterotrophic bacterial community. During the mutualism stage, a self-sufficient nitrogen cycle might contribute to the healthy survival of both *Synechococcus* and heterotrophic bacterial communities in 2 years without any exogenous nutrition supplementation.

Taken together, the interactions among different bacterial members with significantly distinct effects (inhibitory or promotive) on *Synechococcus* growth may determine the overall relationship of the whole heterotrophic bacterial community with *Synechococcus*; the succession of the bacterial community structure during the long-term cocultivation process drove the dynamic changes of its relationship with *Synechococcus* from antagonism to commensalism and then to mutualism; the final mutualistic relationship between *Synechococcus* and the heterotrophic bacterial community allowed them to meet each other’s nutritional needs through the internal self-sufficient nutrient cycle (e.g., the nitrogen cycle), thereby supporting their healthy survival for up to 2 years without any exogenous nutrient supply (Fig. 5). This hints that the ubiquity and competitive advantage of *Synechococcus* in global oceans might also be partially attributed to the contribution of heterotrophic bacterial communities.

## MATERIALS AND METHODS

### Establishment of the cocultivation system of *Synechococcus* and the natural bacterial community.

A model marine cyanobacterial strain, *Synechococcus* sp. PCC7002, was acquired from the Pasteur Culture Collection (53), which was isolated from the coast of the North Atlantic and belongs to subcluster 5.2 of the genus *Synechococcus* (see Fig. S6 in the supplemental material). The strain was streaked onto an A^+^ agar plate (54) and purified repeatedly in solid and liquid media. The sterility of the *Synechococcus* sp. PCC7002 culture was verified routinely and before all experiments by fluorescence microscopy, flow cytometry, agar media, and 16S rRNA gene sequencing (55). Axenic *Synechococcus* was inoculated into fresh A^+^ medium at a 1% volume ratio and cultivated to the exponential growth phase in 14 days at 26°C at 50 μmol m^−2^ s^−1^ under a 12-h-light/12-h-dark (12L:12D) photoperiod. Exponentially growing axenic *Synechococcus* cells were later employed for the experiments described below. For the natural bacterial community, three replicates of surface seawater samples (∼200 ml each) collected from the coast of Qingdao, China (36.06°N, 120.32°E), in summer (August) were incubated in the dark for 72 h (to suppress algal growth) and subjected to sequential filtration through 3.0- and 0.22-μm-pore-size polycarbonate filters (Millipore, Ireland). The 0.22-μm-pore-size filters, which retained the bacterial communities, were dispersed in 20 ml of an exponentially growing axenic *Synechococcus* culture (1.7 × 10^5^ cells ml^−1^). The culture was gently shaken to allow the bacterial assemblage to separate from the filters. Like this, triplicate *Synechococcus-*bacterium cocultures were established along with a control set of triplicate axenic *Synechococcus* cultures in A^+^ medium.

10.1128/mBio.01614-21.7 FIG S6 Phylogenetic tree of 16S rRNA gene sequences of *Synechococcus* strains. Clade and subcluster designations follow the nomenclature of S. Bemal and A. C. Anil (FEMS Microbiol Ecol 92:fiw162, 2016, https://doi.org/10.1093/femsec/fiw162). The tree was constructed by the neighbor-joining method with Muscle, aligning 59 16S rRNA nucleotide sequences of *Synechococcus* strains with 1,000 bootstrap replicates. Download FIG S6, TIF file, 2.1 MB. Copyright © 2021 Zhang et al. 2021 Zhang et al. https://creativecommons.org/licenses/by/4.0/ This content is distributed under the terms of the Creative Commons Attribution 4.0 International license.

### Serial subculturing of the *Synechococcus*-bacterium coculture and subsequent long-term static cocultivation.

The above-described cocultivation systems were incubated at 26°C at 50 μmol m^−2^ s^−1^ under a 12L:12D photoperiod for 7 days (i.e., the first generation of the subculture [1st GS]). Following filtration through a 3.0-μm-pore-size polycarbonate membrane to remove large particles or biological aggregates, 1 ml of the filtrate containing the bacterial assemblage (and also *Synechococcus* cells due to the similar size range, i.e., <3 μm) was transferred to another exponentially growing axenic *Synechococcus* culture (20 ml) to initiate the “second generation” of the subculture (for another 7 days) (2nd GS). The remaining filtrate from the three parallel experimental groups (replicates) was mixed, and the bacteria in the filtrate were retained on 0.22-μm-pore-size membranes, flash-frozen, and preserved at −80°C for bacterial community analysis. Thereafter, subsequent subcultures (generations) were obtained according to the above-described steps until the end of the 20th generation (in total, 140 days of serial subculturing) (Fig. 1a). In addition, considering that viruses (especially cyanophage) and cyanobacteria from the natural seawater may be introduced into the experimental system, the abundance of viruses and the composition of cyanobacteria in the system were tested, and their interference was subsequently excluded (Text S1; see also Table S14 at https://doi.org/10.6084/m9.figshare.15059517.v3).

At the end of the 20th generation (20th GS), the final three replicates of the cocultivation system were mixed and filtered, and the <3-μm filtrate was inoculated into two new culture flasks (at a 1% ratio) containing 3 liters of an exponentially growing *Synechococcus* culture in A^+^ medium. The system was incubated at room temperature at 20°C to 25°C under natural light/dark conditions for more than 2 years (i.e., long-term static cocultivation).

### Response of *Synechococcus* to bacterial communities during serial subculture and subsequent long-term static cocultivation.

To reveal the different responses of *Synechococcus* to bacterial communities during serial subculture and long-term static cocultivation, at the end of each subculture (generation) and 2-year static cocultivation, the maximum quantum yield of photosystem II (*F_v_*/*F_m_*) of *Synechococcus* in the cocultivation systems was measured and compared with that in the axenic control groups as an indicator of the autotroph’s photosynthetic performance. The samples were dark acclimated for 15 min, and the *F_v_*/*F_m_* was analyzed using a dual-wavelength pulse-amplitude-modulated fluorescence monitoring system (Dual-PAM; Heinz Walz, Germany) at a wavelength of 620 nm (56).

### Dynamic changes in bacterial community structure during serial subculture and subsequent long-term static cocultivation.

To understand whether the bacterial community changed dynamically during long-term cocultivation, the bacterial community structure was analyzed according to the method of Picazo et al. (57) in each of the serial subcultures and in the 2-year static cocultivation system. Genomic DNA was extracted using the FastDNA Spin kit (MP Biomedicals). Detailed procedures for sequence analysis are provided in Text S1. Constrained principal-coordinate analysis (PCoA) and hierarchical clustering based on the Bray-Curtis distance matrix were employed to explore the similarities between different bacterial communities. The linear discriminant analysis (LDA) effect size (LEfSe) method (http://huttenhower.sph.harvard.edu/lefse/) was used to identify the most differentially abundant taxa between different groups. The raw reads were deposited in the Genome Sequence Archive of the National Genomics Data Center (NGDC) (58).

### Isolation of bacterial individuals from the cocultivation system.

Individual bacteria were isolated from the cocultivation system using solid marine agar 2216E medium from the 3rd generation of the subculture (3rd GS), which represented the antagonism stage of the relationship, and during long-term static cocultivation, which represented the mutualism stage. In addition, potential nitrogen-fixing bacteria were also isolated using nitrogen-free media (i.e., Jensen’s medium and New Fabian broth [NFB] medium) under oxic and partially oxic conditions (59, 60). A total of 1,264 bacterial colonies were purified and identified (see Tables S1 and S2 at https://doi.org/10.6084/m9.figshare.15059517.v3) via 16S rRNA gene analysis (61). The detailed procedure for bacterial identification is provided in Text S1. The 16S rRNA gene sequences were deposited in GenBank.

### Impacts of the representative bacterial individuals on the growth of *Synechococcus*.

The effects of all 326 different bacterial individuals from the 3rd GS and 34 out of 938 strains representing different species from the long-term static cocultivation system on *Synechococcus* growth were analyzed. Exponentially growing bacteria (with 3 parallel replicates) were washed and resuspended in A^+^ medium to a final concentration of ca. 10^6^ cells ml^−1^. One milliliter of these bacterial suspensions was added to a 10-ml axenic *Synechococcus* (10^6^ cells ml^−1^) culture and incubated at 26°C at 50 μmol m^−2^ s^−1^ under a 12L:12D photoperiod for 20 days. The chlorophyll fluorescence was measured at a fixed time every day using a BioTek Synergy HT plate reader with excitation/emission wavelengths of 440/680 nm to determine the growth status of *Synechococcus*. The following formula was used for the calculation of average changes in florescence values (μ): μ = [ln(*N_t_*) − ln(*N*_0_)]/Δ*t*, where *N_t_* and *N*_0_ are the *Synechococcus* fluorescence values at *t* and 0 days, respectively, and Δ*t* is the incubation time. Significant effects (promotion and inhibition) of bacteria on the growth of *Synechococcus* were determined by a *t* test (*P < *0.05), using μ values between the cocultivation group and the axenic *Synechococcus* group.

### Interactions among members of the heterotrophic bacterial community.

Forty-two representative bacterial strains belonging to different genera or having different impacts on *Synechococcus* from the 3rd GS and 34 strains representing different species from the long-term static cocultivation system were tested for the production of autoinducer-2, a QS molecule, as described previously by Bassler et al. (62). In addition, a plate assay was used to test the actual occurrence of interactions between different bacterial individuals. Pseudomonas sp. syn326 and *Erythrobacter* sp. SN021, representing the most abundant bacterial species from the 3rd GS and the long-term static cocultivation system, respectively, were selected to test their effects on other bacterial strains. The detailed procedures are included in Text S1.

### Determination of inorganic nitrogen in the cocultivation system and the genes related to the nitrogen cycle in the bacterial community.

The inorganic nitrogen (i.e., nitrate, nitrite, and ammonium) concentrations in the cocultivation systems were measured using an AutoAnalyzer (AA3; Bran and Luebbe, Germany).

Metagenomic analysis was employed to detect the genes related to the nitrogen cycle in the 2-year static cocultivation system (see Text S1 for detailed procedures for sequence analysis). Sequence reads were deposited in the NGDC database. Additionally, 34 representative bacterial strains from the 2-year static cocultivation system were screened for the presence of the nitrogenase gene *nifH* using primers PolF and PolR (63, 64). Meanwhile, their potential for nitrogen fixation was further determined by the acetylene reduction assay (Text S1). Moreover, the 34 representative strains from the 2-year static cocultivation system and 42 representative strains from the 3rd GS were also tested for nitrate-reducing ability according to standard approaches (Text S1 and Fig. S7) (65).

10.1128/mBio.01614-21.8 FIG S7 Nitrate-reducing bacteria in different cocultivation systems. The abilities to reduce nitrate to nitrite or nitrogen by the 42 (a) and 34 (b) representative bacterial individuals from the antagonism and mutualism stages, respectively, were tested. Beneficial bacteria for the growth of *Synechococcus* are marked in red, and inhibitory bacteria are in blue. Download FIG S7, TIF file, 2.8 MB. Copyright © 2021 Zhang et al. 2021 Zhang et al. https://creativecommons.org/licenses/by/4.0/ This content is distributed under the terms of the Creative Commons Attribution 4.0 International license.

### Data availability.

The data that support the findings of this study are available from the corresponding author upon reasonable request. Sequence data have been deposited in the NGDC database under accession no. CRA003602 and CRA003605 and in GenBank under accession no. MW409752 to MW410111.
