# Supplementary material for: Long-Term Survival of Synechococcus and Heterotrophic Bacteria without External Nutrient Supply after Changes in Their Relationship from Antagonism to Mutualism
Source: mBio. 2021 Aug 31;12(4):e01614-21. doi: 10.1128/mBio.01614-21 (PMC8406228; doi:10.1128/mBio.01614-21)
Supplement: TEXT S1 [file mbio.01614-21-s0001.doc]

Supplementary Materials for

**Long-term survival of *Synechococcus* and heterotrophic bacteria without external nutrient supply after changes in their relationship from antagonism to mutualism**

Zenghu Zhang†, Shailesh Nair†, Lili Tang, Hanshuang Zhao, Zhenzhen Hu, Mingming Chen, Yao Zhang, Shuh-Ji Kao, Nianzhi Jiao, Yongyu Zhang*

* Corresponding author. Email: zhangyy@qibebt.ac.cn.

† These authors contributed equally to this work.

**This file includes:**

Supplementary Materials and Methods

Supplementary Results

Figures S1-S7 legends

References

**Other Supplementary Material for this manuscript includes the following:**

Table S1-S14 (https://doi.org/10.6084/m9.figshare.15059517.v3)

**Supplementary** **Materials and Methods**

***Collection of natural seawater bacterial community***

The surface seawater samples (replicates) were collected from the coast of Qingdao, China (~200 ml each) using insulated dark 1-L acid-cleaned carboys and kept in cold-dark conditions for 72 hours (to suppress algal growth). Large particles were removed using 3.0-μm polycarbonate filters and the bacterial community was collected on the 0.22-μm membrane filters. Subsequently, parts of the filters that retain bacterial communities were preserved at -80°C for DNA extraction. The other filters were used to establish the *Synechococcus-*bacteria cocultivation systems.

***Microbial counting in the first generation of subculture***

In order to evaluate the impact of potential viruses in the cocultivation system, especially cyanophage, we tested the dynamic changes of *Synechococcus* and virus abundance in the first generation of subculture. Samples (1.5 mL) were collected and fixed with glutaraldehyde (final concentration: 0.5%), flash-frozen in liquid nitrogen, and stored at -80°C. The abundance of *Synechococcus* and virus was determined by flow cytometry according to the description of Liang et al (2016) (1).

***Picoeukaryotic contamination assessment***

Picoeukarotic contamination was checked by epifluorescence microscopy and flow cytometry following standard protocols (2). For epifluorescence microscopy, 1 ml of the sample was serially diluted in sterile artificial seawater, collected on a 0.22 µm pore size black polycarbonate filter, and stained with SYBR Green I. The stained samples were observed under 100x magnifications by epifluorescence microscopy (LEICA DM 2000) under green/blue excitation. For flow cytometry analysis, 1 ml of the collected sample was diluted to 10- to -100 folds with sterile PBS solution and analyzed on a FACSAria II Flow Cytometer (BD Biosciences) with double-distilled water as the sheath fluid, at a flow rate of ~200 cells/s for 60 s. The cells were differentiated based on their size (forward vs side scatter), chlorophyll (PerCP-Cy5.5), phycoerythrin (PE-A), and allophycocyanin (APC-A) pigments.

***DNA extraction, sequencing, and analysis***

DNA was extracted using the FastDNA SPIN kit (MP Biomedicals). The DNA samples were sent to Majorbio Company (Shanghai, China) for sequencing on the Illumina MiSeq Platform. Universal bacterial primers 338F (5’-ACTCCTACGGGAGGCAGCA-3’) and 806R (5’-GGACTACHVGGGTWTCTAAT-3’) were used to amplify the hypervariable V3-V4 region of the 16S rRNA gene, except for DNA samples from the two-year static cocultivation, wherein primers 343F (5’- TACGGRAGGCAGCAG-3’) and 798R (5’-AGGGTATCTAATCCT-3’) were used to amplify the similar region of the 16S rRNA gene. The paired-end reads were merged using FLASH (version 1.2.11) (3) and quality-filtered via Vsearch (version 2.13.6). Resultant data were denoised with the unoise3 command in Usearch (version 10) and the amplicon sequence variants (ASVs) were defined (4). Potential chimeras were removed using reference-based chimera detection in Uchime. Taxonomy was assigned to ASVs against the Silva database (release 132) (5). To eliminate the interference of *Synechococcus*, the corresponding sequences were filtered out and the remaining sequences were normalized by random subsample. Alpha diversity indexes were calculated using the R package “vegan”. Phylogenetic Investigation of Communities by Reconstruction of Unobserved States 2 (PICRUSt2) software package was used to predict the approximate functional potential of the bacterial communities (6).

***Identification of bacterial strains***

Genomic DNA of bacterial strains was extracted using the TIANamp Bacteria DNA kit (TIANGEN). PCR amplification was performed using the primer pair 27F and 1492R (7) under the conditions as follows: denaturation at 95°C for 1 min, annealing at 55°C for 1 min, and extension at 72°C for 1 min 30 seconds with 30 cycles. High-quality 16s rRNA gene sequences were aligned to the sequences deposited in the EzBioCloud database (8)for taxonomic identities. A phylogenetic tree based on the 16S rRNA gene was constructed in MEGA 7.0 (9).

***Bioassay for detection of autoinducer-2 producing ability of bacterial strains***

The reporter strain *Vibrio harveyi* BB170 was cultured in AB medium (10) overnight at 30°C (OD_600_ = 0.8-1.2) and then diluted 1:5000 in fresh AB medium. *V. harveyi* BB150 (positive control) and tested bacterial strains after a 36-60 h growth in 2216E medium were filtered through 0.22-μm filters. These cell-free filtrates and axenic 2216 medium (negative control) were added to diluted reporter strain culture at a ratio of 1:50. The bioluminescence of these mixtures was measured in 7 h after addition. The filtrate of positive strain could stimulate a significantly more intense luminescence in *V. harveyi* BB170 than that of the negative control (*p* < 0.05, t-test).

***Plate assay method for testing the bacteria-bacteria interactions***

Tested bacteria were spread on the 2216E agar plate and an agar slab containing *Pseudomonas* sp. syn326 or *Erythrobacter* sp. SN021 was placed at the center of each plate. The inhibition or promotion zone around the inoculated slab was observed every day during 7-day incubation.

***Acetylene reduction assay for detection of nitrogenase activity***

30 ml of bacterial culture was inoculated into a 125 ml sterile airtight vial in duplicate. 10% of the headspace gas was replaced with pure acetylene. Ethylene concentrations in the vials were measured at 0 and 24 h of incubation at 28°C, using a gas chromatograph equipped with a flame ionization detector (GC-8A, Shimadzu Corp., Kyoto, Japan). Airtight vials (containing 10% acetylene gas) filled with 30 ml sterile distilled water served as controls. A significant increase in the amount of ethylene in the test vials indicated that acetylene has been reduced to ethylene by the tested strain, i.e., the strain has nitrogenase activity.

***Metagenomic sequencing and analysis of the bacterial community in the mutualism stage***

DNA was extracted from the 100 ml culture in the mutualism stage using the FastDNA SPIN kit (MP Biomedicals) and sent to Oebiotech Company (Shanghai, China) for sequencing on an Illumina NovaSeq6000. Quality control was conducted using the KneadData pipeline (https://bitbucket.org/biobakery/kneaddata) with the removal of *Synechococcus* sequences (GenBank accession: GCA_000019485) by bowtie2. Clean reads were aligned to a manually curated database NCycDB for annotation of nitrogen-cycling genes (11). Clean reads were further assembled via SPAdes using default settings with K-mer lengths of 21, 33, 55, 77, 99, 127 (12) and annotated via GhostKOALA (13) for KO orthologs. For phosphorus cycle genes, a related KO ortholog table was built from Liang et. al. 2020 (14) and vitamin B_12_ from KEGG-Decoder (15). Similarly, the contigs were fed into the FeGenie tool to explore Fe-related genes (16). Moreover, clean reads were binned into Metagenome-Assembled Genomes (MAGs) using the MetaWRAP pipeline. MAGs were assessed for genome size, contamination, and completeness using the tool CheckM (17). Genes of MAGs were annotated with Rapid Annotation using the Subsystem Technology SEED viewer (RAST) (18, 19). We focused on genes related to auxin biosynthesis, a typical metabolic activity of heterotrophic bacteria to promote the growth of photosynthetic organisms.

***Sequencing and annotation of Synechococcus sp. PCC7002 genome***

We isolated and sequenced the axenic *Synechococcus* from the mutualism stage. In brief, a single colony of *Synechococcus* sp. PCC7002 was isolated on A+ soft-agar plate. The purity of *Synechococcus* was verified by fluorescence microscopic examination after staining with SYBR Green I. Genomic DNA of the *Synechococcus* isolate was extracted using the TIANamp Bacteria DNA kit (TIANGEN) and sent to Biomarker Technologies Company (Beijing, China) for Whole Genome Sequencing on an Illumina NovaSeq6000. The resultant sequences were trimmed and quality checked via the KneadData pipeline (https://bitbucket.org/biobakery/kneaddata). The clean sequences were assembled into contigs via SPADES 3.14.1 with default settings (20) and functional annotation was assigned via GHOSTKOALA (http:// www.kegg.jp/ghostkoala/). Predicted pathway completion was determined via KEGG-Decoder (15). The same sequencing and annotation process was also applied to the initial axenic *Synechococcus* sp. PCC7002. The genomic comparison method (21) was used to verify the similarity between the repurified *Synechococcus* from the co-culture and the original axenic *Synechococcus*, with two replicates for each strain. The sequences have been deposited in the NCBI Sequence Read Archive with accession number PRJNA725122.

**Supplementary Results**

***The absence of cyanophage infection and other phytoplankton pollution in the cocultivation system***

In the cocultivation system at the antagonism stage, when the abundance of *Synechococcus* decreased significantly, no obvious change in virus abundance was observed (Fig. S1). This indicates that the decrease in the abundance of *Synechococcus* was not due to virus infection.

Except for the *Synechococcus* sp. PCC7002, no other phytoplankton was observed in the cocultivation system by fluorescence microscopy and flow cytometry method. Meanwhile, the purity of *Synechococcus* sp. PCC7002 in the cocultivation system was further confirmed by the 16s rDNA analysis and genomic comparison (Table S14).

***Nitrate-reducing bacteria existed in different cocultivation systems***

Forty-two representative bacterial strains from the antagonism stage and the representative strains of all 34 different species from the mutualism stage were tested for nitrate reduction ability. 22 and 9 strains from the antagonism and mutualism stages, respectively, were observed to have the ability to reduce nitrate to nitrite. Moreover, 3 strains from the antagonism stage and 9 strains from the mutualism stage could reduce nitrate to nitrogen (Fig. S7).

***Nitrogen-cycling genes annotated in Synechococcus genome***

Axenic *Synechococcus* sp. PCC7002 from the mutualism stage was checked for the presence of potential nitrogen-cycling genes. *Synechococcus* genome contains the genes related to assimilatory nitrate reduction (NarB and NirA), but has no any genes related to dissimilatory nitrate reduction, denitrification, nitrification, and nitrogen fixation (Table S7 and S8).

***The phosphorus, iron, and vitamin B_12_ metabolic genes in the metagenome***

The bacterial metagenome showed the presence of genes for bioavailable phosphorus generation (e.g., the genes involved in organic phosphorus mineralization and solubilization of recalcitrant phosphorus. Table S11). Meanwhile, genes for iron siderophores synthesis, Fe acquisition and transport (Table S12) and the genes for vitamin B_12_ synthesis were also observed (Table S13).

**Figure S1** Time course of virus and *Synechococcus* abundances in the first generation of subculture.

**Figure S2** The distribution of quorum-sensing genes in each cocultivation system. PICRUSt2 was applied for the predictions of microbial functional genes.

**Figure S3** Bacteria-bacteria interactions. Inhibition zone around the agar slab containing *Pseudomonas* sp. syn326 (blue arrow) or *Erythrobacter* sp. SN021 (black arrow) indicated their inhibitory effect on the growth of the tested strains. Promotion zone indicates the beneficial effect.

**Figure S4** Genes involved in the nitrogen cycle in the heterotrophic bacterial community cocultured with *Synechococcus* during the mutualism stage. The numbers in the brackets beside each gene represent the abundance for the gene or gene family.

**Figure S5** Dynamic changes in the bacterial community structure at the phylum level during long-term cocultivation with *Synechococcus*.

**Figure S6** Phylogenetic tree of 16S rRNA gene sequences of *Synechococcus* strains. Clade and sub-cluster designations follow the nomenclature of Bemal et. al, 2016 (22). The tree was constructed by neighbour-joining method by Muscle aligning 59 16s rRNA nucleotide sequences of *Synechococcus* strains with 1000 bootstrap replicates.

**Figure S7** Nitrate-reducing bacteria in different cocultivation systems. The abilities to reduce nitrate to nitrite or nitrogen by the 42 (a) and 34 (b) representative bacterial individuals from the antagonism and mutualism stages respectively were tested. Beneficial bacteria for the growth of *Synechococcus* were marked in red, and inhibitory bacteria were in blue.

**References:**

1. Liang Y, Zhang Y, Yao Z, Luo T, Rivkin RB, Jiao N. 2016. Distributions and relationships of virio-and picoplankton in the epi-, meso- and bathypelagic zones of the Western Pacific Ocean. FEMS Microbiol Ecol 93:fiw23.

2. Metz S, dos Santos AL, Berman MC, Bigeard E, Licursi M, Not F, Lara E, Unrein F. 2019. Diversity of photosynthetic picoeukaryotes in eutrophic shallow lakes as assessed by combining flow cytometry cell-sorting and high throughput sequencing. bioRxiv https://doi.org/10.1101/551598.

3. Magoč T, Salzberg SL. 2011. FLASH: fast length adjustment of short reads to improve genome assemblies. Bioinformatics 27:2957–2963.

4. Edgar RC. 2016. UNOISE2: improved error-correction for Illumina 16S and ITS amplicon sequencing. bioRxiv 81257.

5. Quast C, Pruesse E, Yilmaz P, Gerken J, Schweer T, Yarza P, Peplies J, Glöckner FO. 2013. The SILVA ribosomal RNA gene database project: Improved data processing and web-based tools. Nucleic Acids Res 41.

6. Douglas GM, Maffei VJ, Zaneveld J, Yurgel SN, Brown JR, Taylor CM, Huttenhower C, Langille MGI. 2020. PICRUSt2: An improved and customizable approach for metagenome inference. bioRxiv 672295.

7. Weisburg WG, Barns SM, Pelletier DA, Lane DJ. 1991. 16S ribosomal DNA amplification for phylogenetic study. J Bacteriol 173:697–703.

8. Yoon S-H, Ha S-M, Kwon S, Lim J, Kim Y, Seo H, Chun J. 2016. Introducing EzBioCloud: A taxonomically united database of 16S rRNA and whole genome assemblies. Int J Syst Evol Microbiol 67:1613–1617.

9. Kumar S, Stecher G, Tamura K. 2016. MEGA7: Molecular evolutionary genetics analysis version 7.0 for bigger datasets. Mol Biol Evol 33:1870–1874.

10. Bassler BL, Wright M, Showalter RE, Silverman MR. 1993. Intercellular signalling in *Vibrio harveyi*: sequence and function of genes regulating expression of luminescence. Mol Microbiol 9:773–786.

11. Tu Q, Lin L, Cheng L, Deng Y, He Z. 2019. NCycDB: A curated integrative database for fast and accurate metagenomic profiling of nitrogen cycling genes. Bioinformatics 35:1040–1048.

12. Nurk S, Meleshko D, Korobeynikov A, Pevzner PA. 2017. MetaSPAdes: A new versatile metagenomic assembler. Genome Res 27:824–834.

13. M K, Y S, K M. 2016. BlastKOALA and GhostKOALA: KEGG tools for functional characterization of genome and metagenome sequences. J Mol Biol 726–731.

14. Liang JL, Liu J, Jia P, Yang T tao, Zeng Q wei, Zhang S chang, Liao B, Shu W sheng, Li J tian. 2020. Novel phosphate-solubilizing bacteria enhance soil phosphorus cycling following the ecological restoration of land degraded by mining. ISME J 14:1600–1613.

15. Graham ED, Heidelberg JF, Tully BJ. 2018. Potential for primary productivity in a globally-distributed bacterial phototroph. ISME J 12:1861–1866.

16. Garber AI, Nealson KH, Okamoto A, McAllister SM, Chan CS, Barco RA, Merino N. 2020. FeGenie: a comprehensive tool for the identification of iron genes and iron gene neighborhoods in genome and metagenome assemblies. Front Microbiol 11.

17. Parks DH, Imelfort M, Skennerton CT, Hugenholtz P, Tyson GW. 2015. CheckM: assessing the quality of microbial genomes recovered from isolates, single cells, and metagenomes. Genome Res 25:1043–1055.

18. Overbeek R, Begley T, Butler RM, Choudhuri J V, Chuang HY, Cohoon M, de Crécy-Lagard V, Diaz N, Disz T, Edwards R, Fonstein M, Frank ED, Gerdes S, Glass EM, Goesmann A, Hanson A, Iwata-Reuyl D, Jensen R, Jamshidi N, Krause L, Kubal M, Larsen N, Linke B, McHardy AC, Meyer F, Neuweger H, Olsen G, Olson R, Osterman A, Portnoy V, Pusch GD, Rodionov DA, Rückert C, Steiner J, Stevens R, Thiele I, Vassieva O, Ye Y, Zagnitko O, Vonstein V. 2005. The subsystems approach to genome annotation and its use in the project to annotate 1000 genomes. Nucleic Acids Res2005/10/11. 33:5691–5702.

19. Aziz RK, Bartels D, Best AA, DeJongh M, Disz T, Edwards RA, Formsma K, Gerdes S, Glass EM, Kubal M, Meyer F, Olsen GJ, Olson R, Osterman AL, Overbeek RA, McNeil LK, Paarmann D, Paczian T, Parrello B, Pusch GD, Reich C, Stevens R, Vassieva O, Vonstein V, Wilke A, Zagnitko O. 2008. The RAST server: rapid annotations using subsystems technology. BMC Genomics2008/02/12. 9:75.

20. Nurk S, Bankevich A, Antipov D, Gurevich AA, Korobeynikov A, Lapidus A, Prjibelski AD, Pyshkin A, Sirotkin A, Sirotkin Y, Stepanauskas R, Clingenpeel SR, Woyke T, McLean JS, Lasken R, Tesler G, Alekseyev MA, Pevzner PA. 2013. Assembling single-cell genomes and mini-metagenomes from chimeric MDA products. J Comput Biol. 20:714–737.

21. Meier-Kolthoff JP, Auch AF, Klenk H-P, Göker M. 2013. Genome sequence-based species delimitation with confidence intervals and improved distance functions. BMC Bioinformatics 14:60.

22. Bemal S, Anil AC. 2016. Genetic and ecophysiological traits of *Synechococcus* strains isolated from coastal and open ocean waters of the Arabian Sea. Fems Microbiol Ecol, 92: fiw162.
