# Supplementary figures and images for: Long-Term Survival of Synechococcus and Heterotrophic Bacteria without External Nutrient Supply after Changes in Their Relationship from Antagonism to Mutualism
Source: mBio. 2021 Aug 31;12(4):e01614-21. doi: 10.1128/mBio.01614-21 (PMC8406228; doi:10.1128/mBio.01614-21)

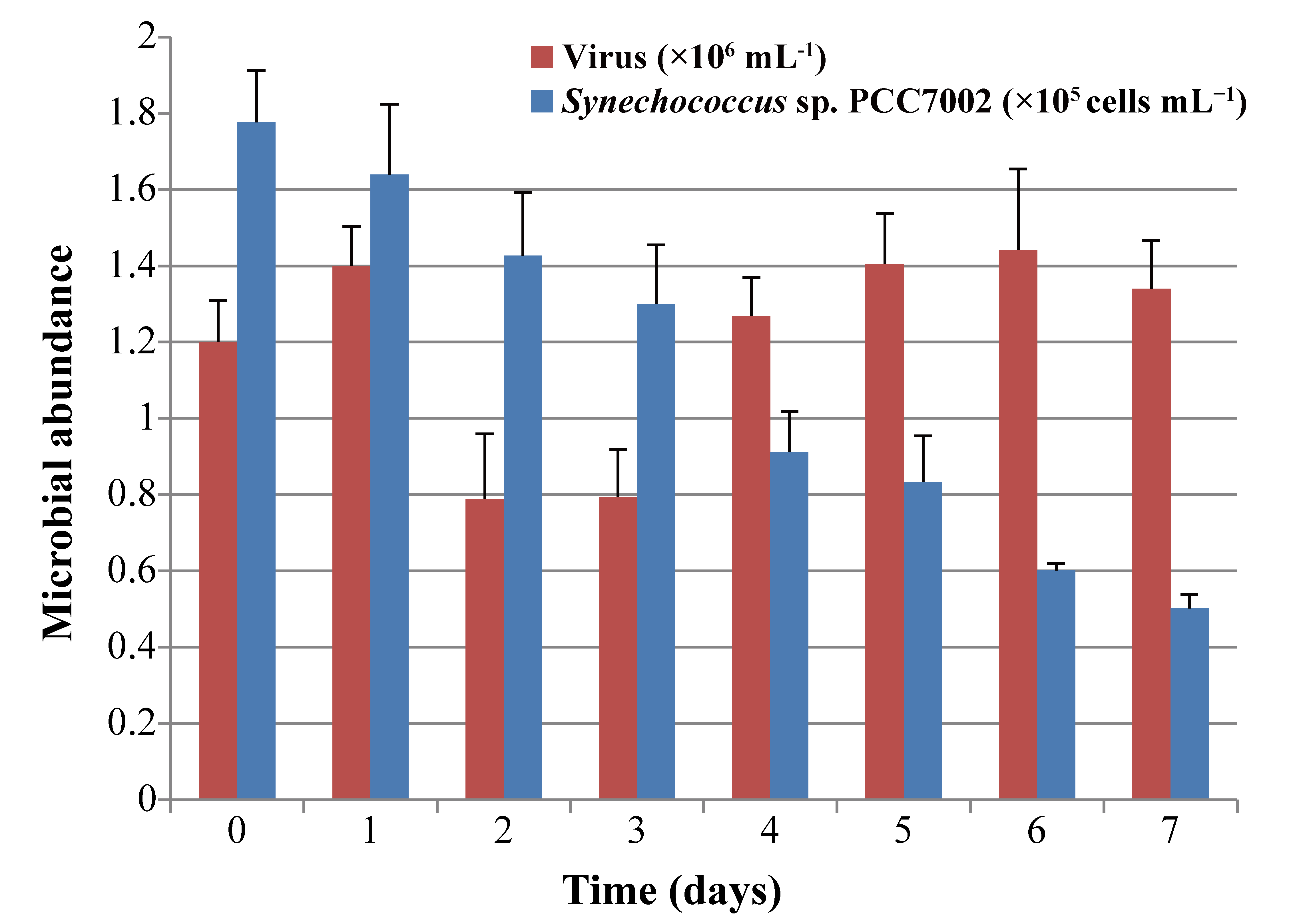

Supplement: FIG S1 [file mbio.01614-21-sf001.tif]

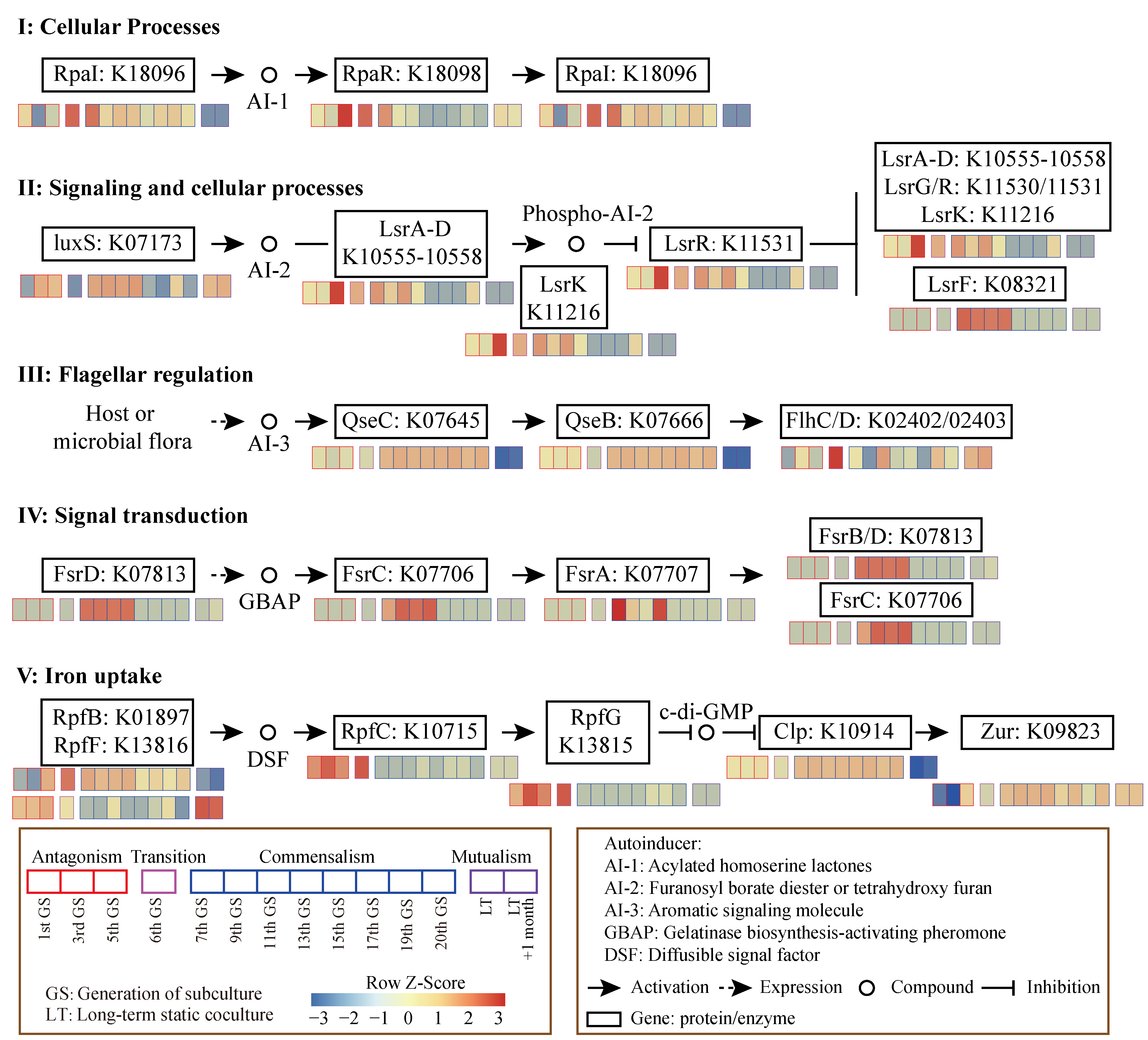

Supplement: FIG S2 [file mbio.01614-21-sf002.tif]

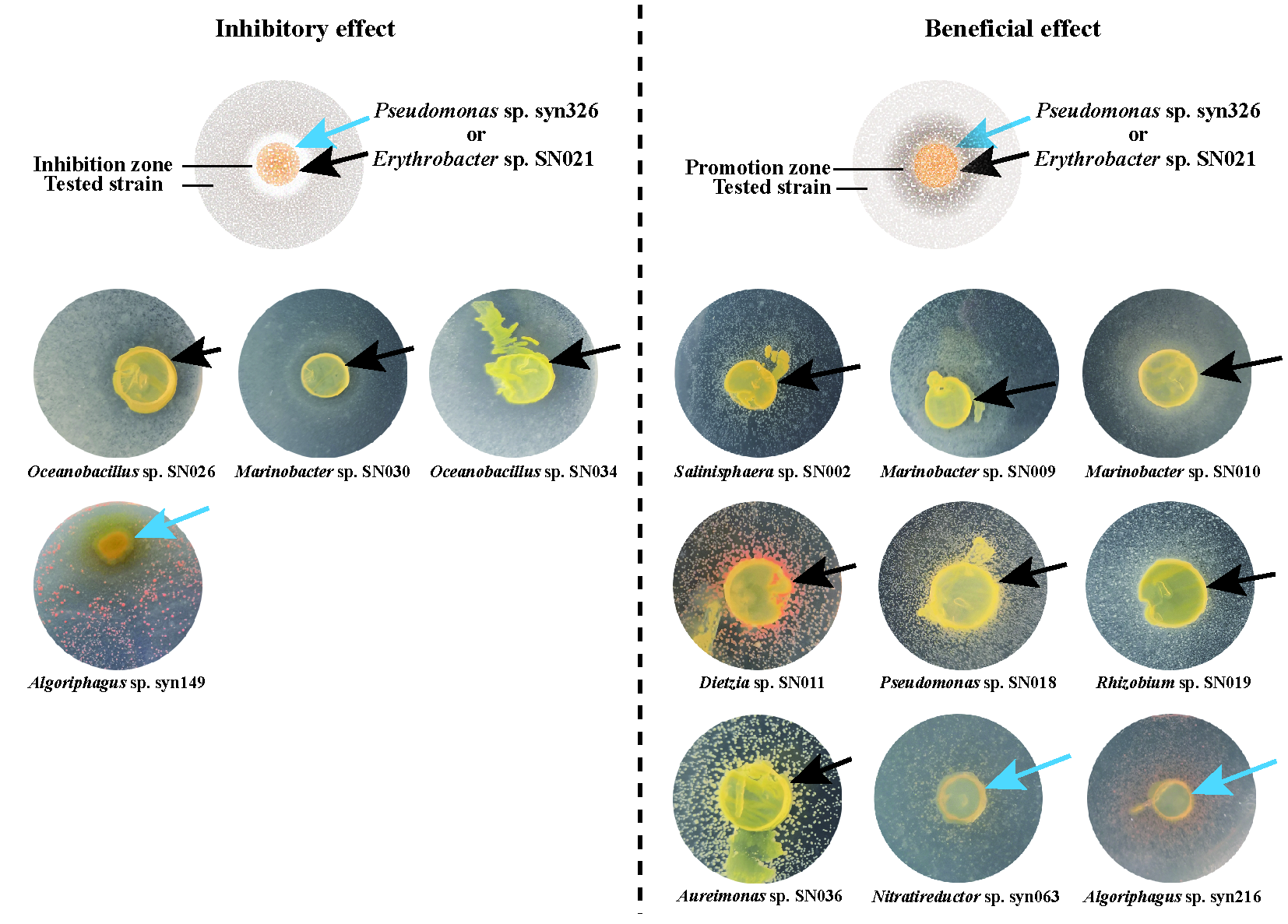

Supplement: FIG S3 [file mbio.01614-21-sf003.tif]

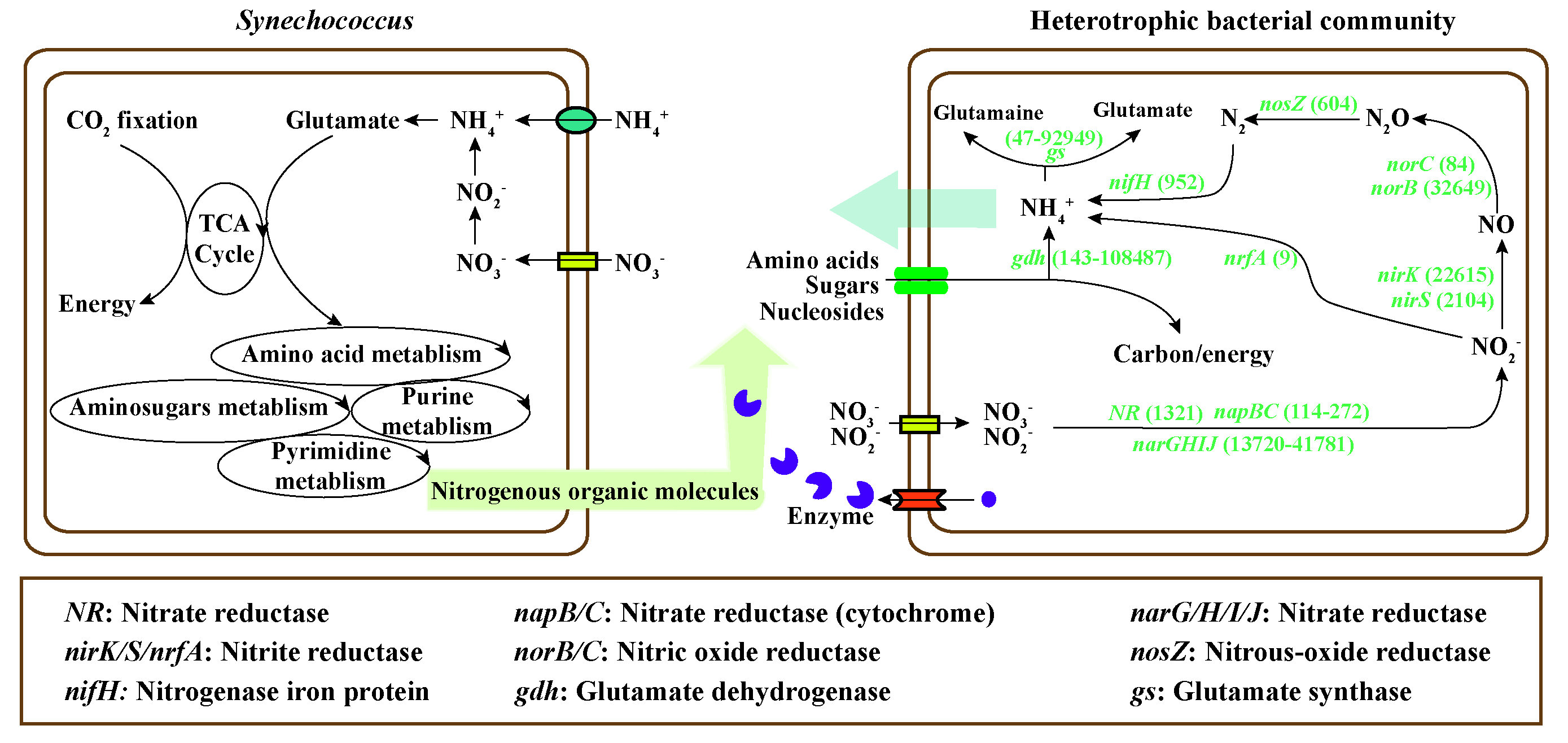

Supplement: FIG S4 [file mbio.01614-21-sf004.tif]

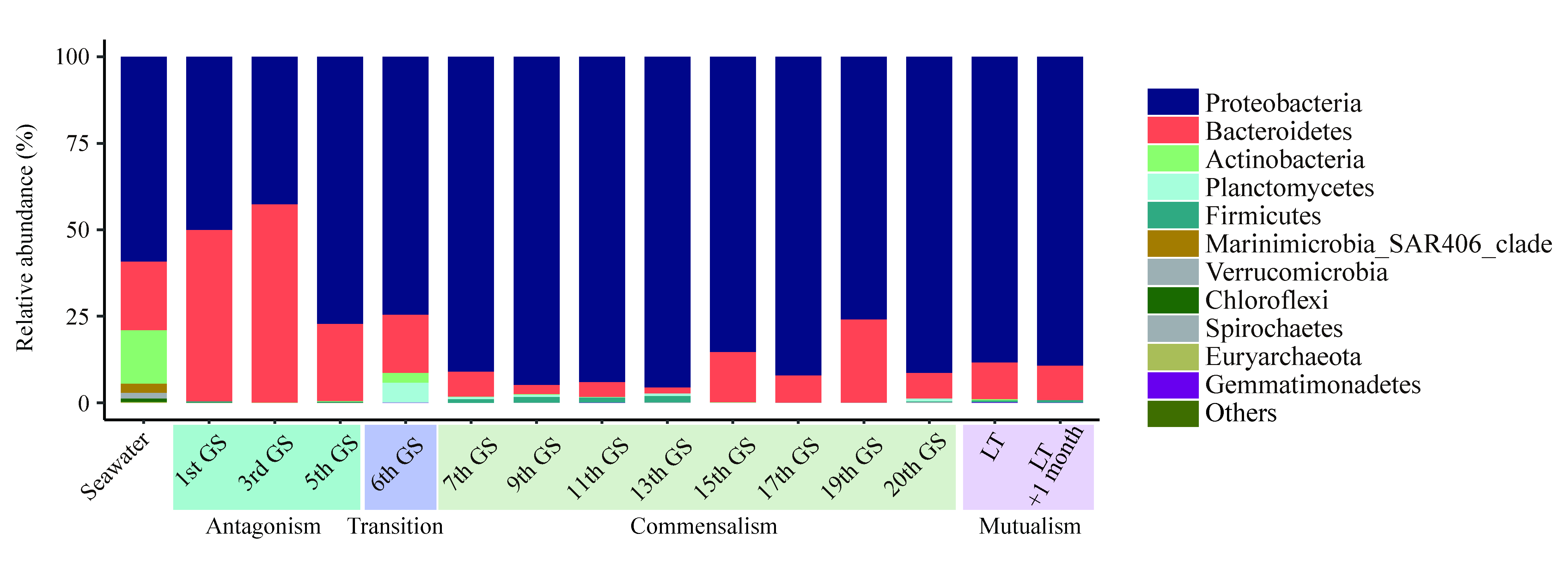

Supplement: FIG S5 [file mbio.01614-21-sf005.tif]

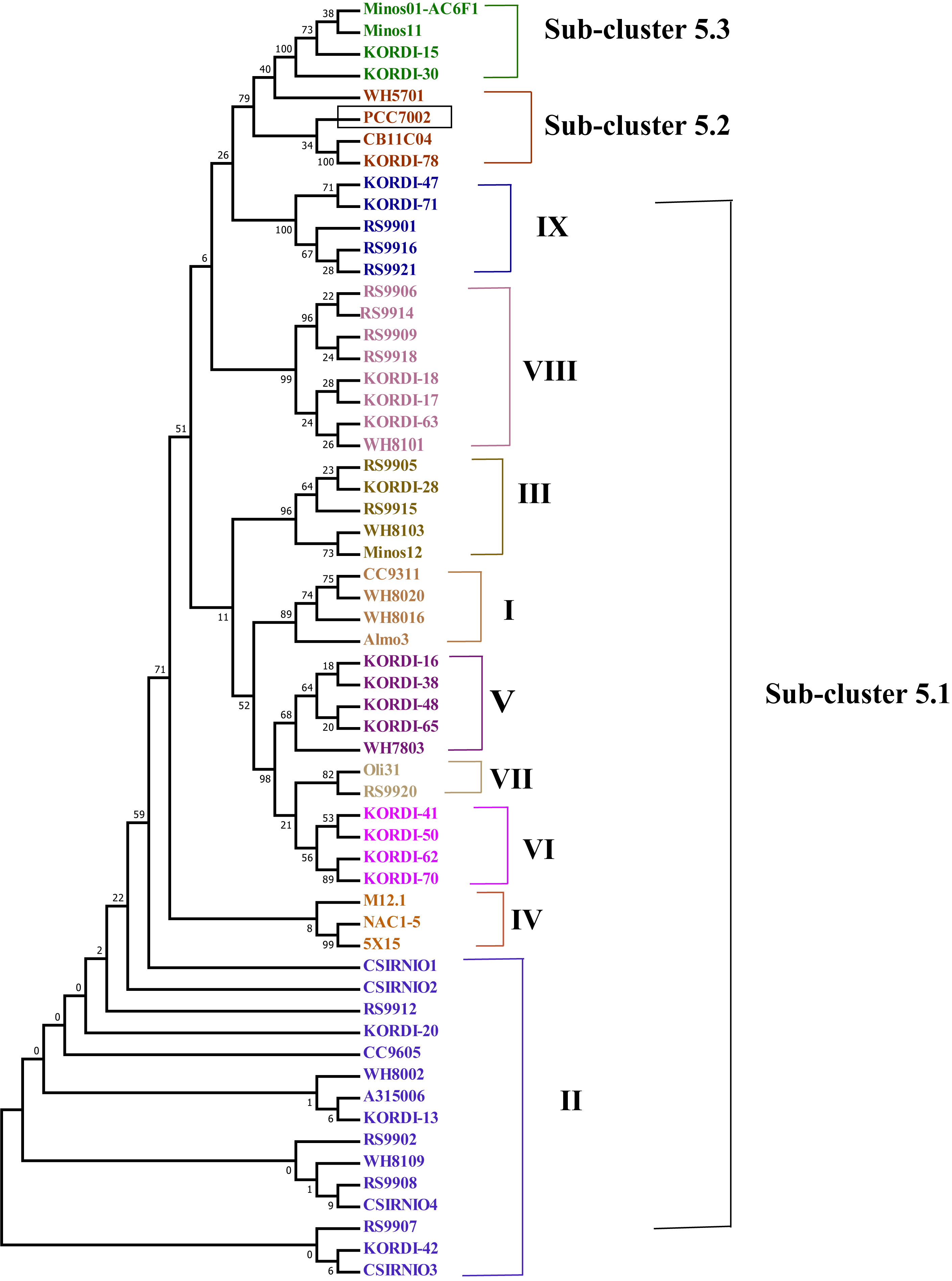

Supplement: FIG S6 [file mbio.01614-21-sf006.tif]

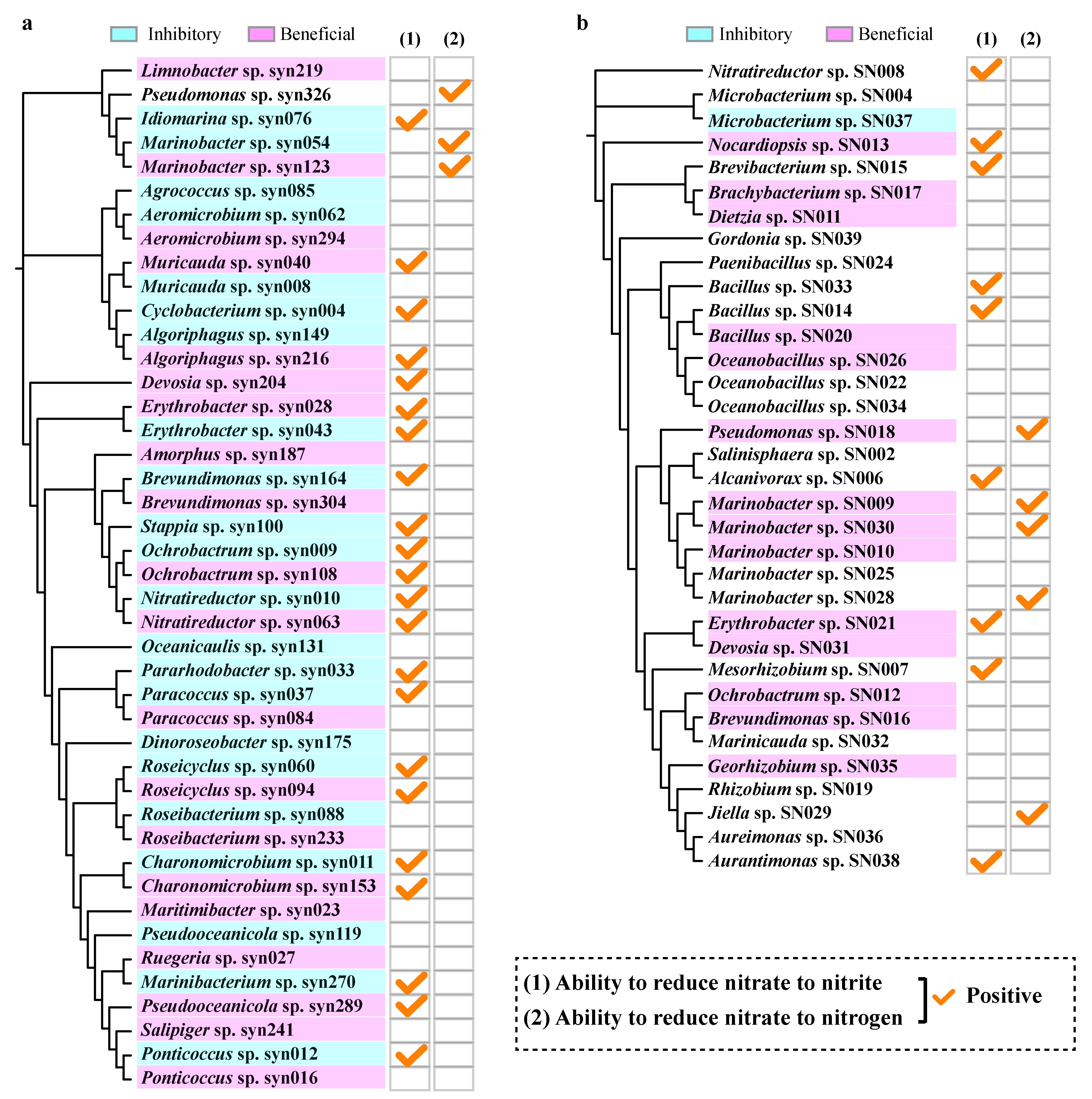

Supplement: FIG S7 [file mbio.01614-21-sf007.tif]
